# Allograft function predicts mortality in kidney transplant recipients with severe COVID-19: a paradoxical risk factor

**DOI:** 10.3389/fimmu.2024.1335148

**Published:** 2024-02-13

**Authors:** Han Luo, Jingyu Wen, Hongji Yang, Qing Ran, Yifu Hou

**Affiliations:** ^1^ Department of Organ Transplantation, Sichuan Provincial Peoples Hospital, University of Electronic Science and Technology of China, Chengdu, China; ^2^ School of Medicine, University of Electronic Science and Technology of China, Chengdu, China; ^3^ Department of Medical Insurance, Sichuan Provincial Peoples Hospital, University of Electronic Science and Technology of China, Chengdu, China; ^4^ Clinical Immunology Translational Medicine Key Laboratory of Sichuan Province & Organ Transplantation Center, Sichuan Academy of Medical Sciences and Sichuan Provincial Peoples Hospital, Chengdu, China

**Keywords:** kidney transplant recipient, allograft function, estimated glomerular filtration rate, creatinine, severe COVID-19

## Abstract

**Introduction:**

Kidney transplant recipients (KTRs) are at a higher risk of severe coronavirus disease (COVID-19) because of their immunocompromised status. However, the effect of allograft function on the prognosis of severe COVID-19 in KTRs is unclear. In this study, we aimed to analyze the correlation between pre-infection allograft function and the prognosis of severe COVID-19 in KTRs.

**Methods:**

This retrospective cohort study included 82 patients who underwent kidney transplantation at the Sichuan Provincial Peoples Hospital between October 1, 2014 and December 1, 2022 and were diagnosed with severe COVID-19. The patients were divided into decreased eGFR and normal eGFR groups based on the allograft function before COVID-19 diagnosis (n=32 [decreased eGFR group], mean age: 43.00 years; n=50 [normal eGFR group, mean age: 41.88 years). We performed logistic regression analysis to identify risk factors for death in patients with severe COVID-19. The nomogram was used to visualize the logistic regression model results.

**Results:**

The mortality rate of KTRs with pre-infection allograft function insufficiency in the decreased eGFR group was significantly higher than that of KTRs in the normal eGFR group (31.25% [10/32] vs. 8.00% [4/50], *P*=0.006). Pre-infection allograft function insufficiency (OR=6.96, 95% CI: 1.4633.18, *P*=0.015) and maintenance of a mycophenolic acid dose >1500 mg/day before infection (OR=7.59, 95% CI: 1.0853.20, *P*=0.041) were independent risk factors, and the use of nirmatrelvir/ritonavir before severe COVID-19 (OR=0.15, 95% CI: 0.030.72, *P*=0.018) was a protective factor against death in severe COVID-19.

**Conclusions:**

Pre-infection allograft function is a good predictor of death in patients with severe COVID-19. Allograft function was improved after treatment for severe COVID-19, which was not observed in patients with non-severe COVID-19.

## 1 Introduction

The coronavirus disease (COVID-19) pandemic continues to pose a significant health risk to people worldwide (1), particularly kidney transplant recipients (KTRs) who are at a higher risk of severe COVID-19 because of their immunocompromised status. Allograft function (AF) plays an important role in severe COVID-19 in KTRs. A study conducted in Spain demonstrated that impaired AF increased the risk of intensive care unit admission and was a predictor of mortality (2), and it is important to determine whether this similar effect is observed on severe COVID-19. Notably, the kidney is an angiotensin-converting enzyme 2 (ACE2) receptor organ (3, 4), causing it to have a high affinity for severe acute respiratory syndrome coronavirus 2 (SARS-CoV-2), the virus that causes COVID-19 (5). The kidney-lung crosstalk theory suggests that lung and kidney damage mutually worsen each others function (6, 7). However, the effect of AF on the prognosis of severe COVID-19 in KTRs remains unclear. Therefore, investigating the prognostic role of pre-infection AF in severe COVID-19 among KTRs is crucial.

Another point of concern is the impact of severe COVID-19 on AF. On the one hand, severe COVID-19 is associated with an increased risk of acute kidney injury (AKI) (8), with a total incidence rate of up to 8% (9). The occurrence of AKI in severe COVID-19 involves various mechanisms, including a systemic inflammatory response, viral infection of renal cells, and severe hemodynamic changes in the kidneys (10), which may damage AF. The incidence of AKI is significantly higher in KTRs than in the general population (11, 12). On the other hand, severe COVID-19 often requires immunosuppressant (IS) drug discontinuation, which increases the risk of subsequent acute rejection (AR) and impairs AF. In addition, the potential effects of small-molecule antivirals, such as nirmatrelvir/ritonavir, on AF during treatment are poorly understood. The question on whether nirmatrelvir/ritonavir exacerbates AF impairment, particularly in patients with impaired AF before infection, requires urgent attention.

In this study, we aimed to utilize AF before, during, and after SARS-CoV-2 infection as indicators to explore the relationship between pre-infection AF and the outcome of severe COVID-19 and determine the factors influencing functional changes in AF during and after infection. We hope to offer valuable insights for future clinical decision-making.

## 2 Materials and methods

### 2.1 Study design and patients

In this retrospective cohort study, we included KTRs who underwent kidney transplantation at the Sichuan Provincial Peoples Hospital between October 1, 2014 and December 1, 2022 and were diagnosed with severe COVID-19. KTRs who died or had renal allograft loss before December 1, 2022 or during the follow-up were excluded. Based on the prevailing period of COVID-19 wave from December 1, 2022 to early February 2023, our follow-up started on December 1, 2022 and ended on April 1, 2023 or at the time of death, whichever came first. According to the 10th Trial Edition of the Guidelines for the Diagnosis and Treatment of COVID-19 (13), severe COVID-19 was diagnosed when adults meet any of the following conditions that cannot be explained for reasons other than COVID-19: 1) shortness of breath or respiratory rate ≥30 times/min; 2) oxygen saturation ≤93% at rest; 3) arterial partial pressure of oxygen/oxygen uptake concentration ≤300 mmHg (1 mmHg=0.133 kPa), and 4) gradual aggravation of clinical symptoms and lung imaging showing significant lesion progression (>50%) within 24-48 h. Based on the AF before COVID-19 diagnosis, AF insufficiency is defined as estimated glomerular filtration rate (eGFR) <60 mL/min, according to the KDIGO guidelines (14). We then divided the patients into decreased eGFR and normal eGFR group, with a cutoff value of estimated glomerular filtration rate <60 mL/min or ≥60 mL/min, respectively.

Kidney allografts from living or deceased organ donors who met the ethical guidelines for kidney donation were used. None of the KTRs received organs from executed prisoners or other institutionalized individuals. This study adhered to the tenets of the Declaration of Helsinki and was approved by the Ethics Committee of the Sichuan Provincial Peoples Hospital (No. 20220901).

### 2.2 Data collection and follow-up

Baseline characteristics of the KTRs, including age, sex, body mass index (BMI), donor type, human leukocyte antigen (HLA) mismatch, primary kidney disease, and comorbidities, were obtained from a scientific registry of the kidney transplantation system (https://www.csrkt.org.cn/door/index). Medical history was obtained through in-patient information collection, outpatient services, online outpatient services, and telephone follow-ups. Clinical data were obtained from medical records, including creatinine (Cr) values 6 months before and 1 and 2 months after infection, vaccination, IS regimen, hospitalization, and COVID-19-related treatment.

### 2.3 Treatment of severe COVID-19

Oral IS medications were discontinued in all patients. The general treatment for COVID-19 included ensuring adequate energy and nutrient intake and paying attention to water and electrolyte balance. The principle of supportive treatment for severe COVID-19 involved actively preventing and treating complications, treating basic diseases, preventing secondary infections, and providing timely organ function support. Respiratory support treatments were selected based on the patients oxygenation index, including nasal catheter or mask oxygen inhalation (PaO_2_/FiO_2_ <300 mmHg), nasal high-flow oxygen therapy, noninvasive ventilation (PaO_2_/FiO_2_ <200 mmHg), invasive mechanical ventilation (PaO_2_/FiO_2_ <150 mmHg), and oxygen therapy during resuscitation to target SpO_2_ ≥94% in KTRs with emergency signs. Once the patient was stable, SpO_2_ >90% was targeted. Airway management and sputum discharge were facilitated to maintain airway patency.

### 2.4 Small-molecule antivirals

Nirmatrelvir/ritonavir (Pfizer, USA), azvudine (Henan Zhenzhen Biotechnology, China), and molnupiravir (Merck, USA) are recommended by the National Health Commission for COVID-19 treatment. Ritonavir, a component of nirmatrelvir/ritonavir, is a potent inhibitor of cytochrome P450 3A and P-glycoproteins. After obtaining informed consent from the patients, azvudine and nirmatrelvir/ritonavir were administered to treat KTRs infected with severe COVID-19. Therapeutic drug monitoring was continued during nirmatrelvir/ritonavir treatment, and the restart dose after nirmatrelvir/ritonavir treatment was adjusted accordingly.

### 2.5 Outcomes

The primary outcome was all-cause mortality, defined as mortality from various causes during the study period. Other outcomes were mainly related to allograft complications. AR is defined as the rapid deterioration of function caused by specific pathological changes in the allograft and can be divided into acute T cell-mediated rejection (TCMR) and acute antibody-mediated rejection (ABMR). BK polyomavirus (BKPyV) infection is mostly covert; however, its reactivation can occur in patients with impaired immune function, eventually leading to BKPyV-associated nephropathy (BKPyVAN). HLA is closely related to functioning of the human immune system and is an important antigenic substance in transplant rejection. Donor-specific antibodies (DSA) are specific antibodies the recipient produces after organ/tissue transplantation against donor tissue antigens, including HLA and non-HLA antibodies.

### 2.6 Statistical analysis

Continuous variables are presented as median and interquartile intervals (IQRs) and were analyzed using a t-test or MannWhitney U test. Categorical variables are reported as frequency counts and percentages and were evaluated using the chi-squared or Fisher exact test. Multivariate logistic regression analysis was used to identify the risk factors for death due to severe COVID-19. The results are reported as odds ratios (ORs), 95% confidence intervals (CIs), and *P*-values. Cox regression was used to construct the final nomogram prediction model. Statistical analyses were performed using GraphPad Prism 8.0 and R version 4.0.3. All tests were two-tailed, and *P*-values <0.05 were considered statistically significant.

## 3 Results

### 3.1 Demographic and clinical characteristics

**Figure 1:**
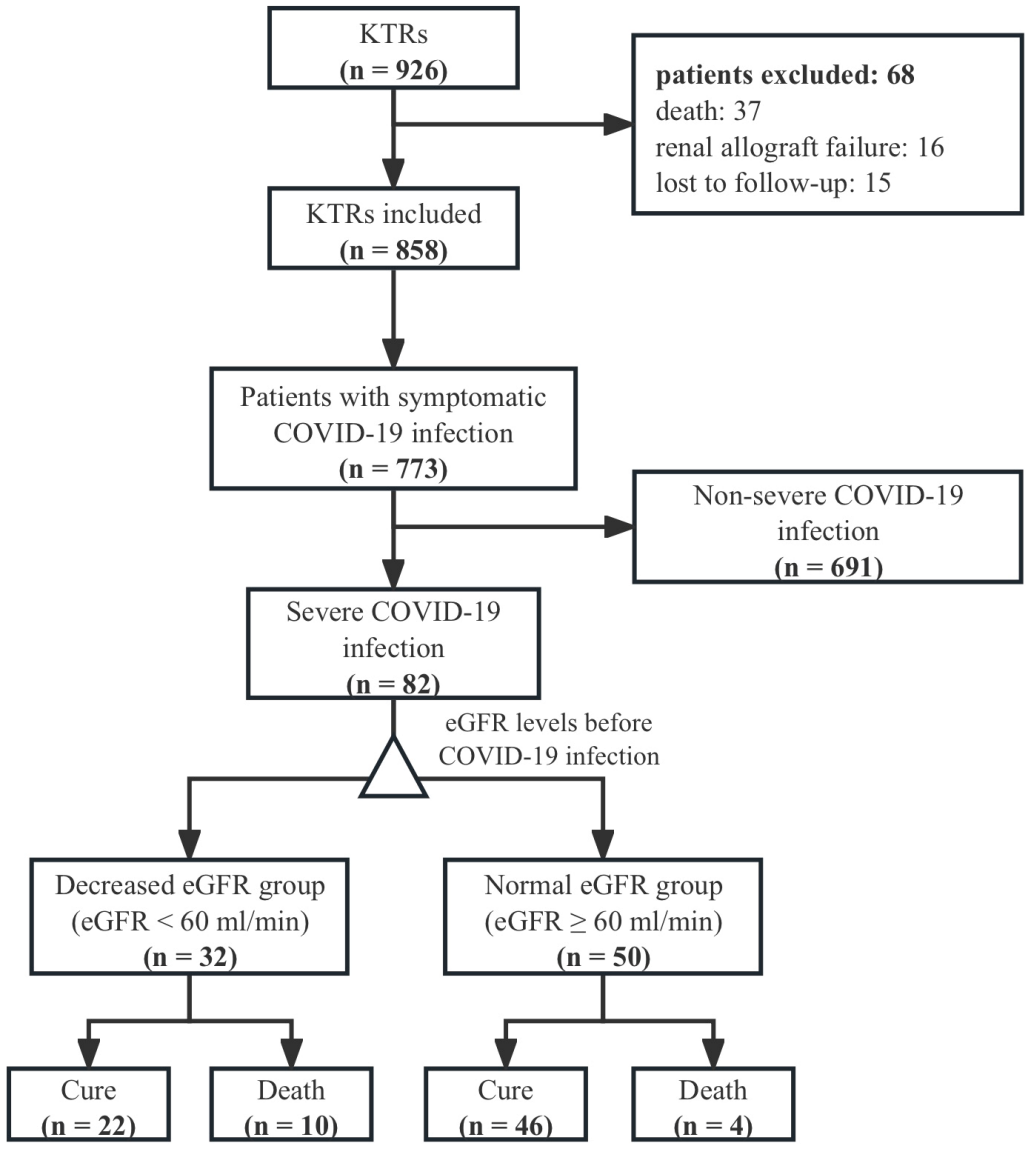
Consort flow diagram of patients.

In total, 926 patients underwent kidney transplantation between October 1, 2014 and December 1, 2022, and 82 KTRs were included in this study. Of these, 32 were in the decreased eGFR group, and 50 were in the normal eGFR group (**Figure 1**), and we compared the baseline characteristics between the groups (**Table 1**). The mean age of patients in the decreased eGFR and normal eGFR group was 43.00 ± 10.6 years and 41.88 ± 8.72 years, respectively (*P*=0.60). Compared with that in the decreased eGFR group, the proportion of complete vaccination was significantly higher in the normal eGFR group (7.32% [6/82] vs. 9.76% [8/82], *P*=0.047). The proportion of mortality was significantly higher in the decreased eGFR group than in the normal eGFR group (31.25% [10/32] vs. 8.00% [4/50], *P*=0.006). However, no significant differences were observed in the patients ages, sexes, comorbidities, primary disease, HLA mismatch, vaccine doses, induction agents, IS regimen adjustment, and donor type between the decreased eGFR and normal eGFR groups.

**Table 1:** Baseline characteristics of patients in the decreased eGFR and normal eGFR groups.

Characteristics	Decreased eGFR group (n=32)	Normal eGFR group (n=50)	*P*-value
Age (years), mean ± SD	43.00 ± 10.61	41.88 ± 8.72	0.60
Sex (male), n (%)	25 (78.13%)	34 (68.00%)	0.32
BMI (kg/m^2^), median (IQR)	21.85 (20.56, 22.87)	21.82 (20.72, 24.12)	0.70
Time since transplantation to end of follow-up (months), median (IQR)	43.63 ± 22.48	53.14 ± 25.73	0.09
Donor type, n (%)			0.39
Deceased	23 (71.87%)	40 (80.00%)	
Living	9 (28.13%)	10 (20.00%)	
HLA mismatch, n (%)			0.12
02	3 (9.37%)	8 (16.00%)	
34	19 (59.38%)	18 (36.00%)	
56	10 (31.25%)	24 (48.00%)	
Primary disease, n (%)			0.59
Focal segmental glomerulosclerosis	1 (3.13%)	0 (0.0%)	
IgA nephropathy	4 (12.50%)	6 (12.00%)	
Diabetes nephropathy	1 (3.13%)	6 (12.00%)	
Nephrotic syndrome	14 (43.75%)	19 (38.00%)	
Chronic glomerulonephritis	7 (21.88%)	8 (16.00%)	
Lupus nephritis	1 (3.13%)	1 (2.00%)	
Unknown	4 (12.50%)	10 (20.00%)	
Comorbidities, n (%)			
Cardiovascular disease	5 (15.63%)	9 (18.00%)	0.78
Chronic lung disease	1 (3.13%)	1 (2.00%)	1.00
Diabetes	8 (25.00%)	14 (28.00%)	0.76
HBV infection, n (%)	11 (34.38%)	17 (34.00%)	0.97
HCV infection, n (%)	5 (15.63%)	9 (18.00%)	0.78
COVID-19 vaccine doses before December 1, 2022			*
0	26 (81.25%)	37 (74.00%)	
1	0 (0.0%)	5 (10.00%)	
2	4 (12.50%)	1 (2.00%)	
3	2 (6.25%)	7 (14.00%)	
Induction agent, n (%)			0.52
IL-2	21 (65.62%)	29 (58.00%)	
r - ATG	10 (31.25%)	15 (30.00%)	
IL-2/r - ATG combined with RTX	0 (0.0%)	2 (4.00%)	
None	1 (3.13%)	4 (8.00%)	
IS before COVID-19 infection, n (%)			1.00
FK plus MPA	28 (87.50%)	45 (90.00%)	
CsA plus MPA	4 (12.50%)	5 (10.00%)	
IS reduction after COVID-19 infection, n (%)			0.36
MPA	1 (3.13%)	5 (10.00%)	
CNI	1 (3.13%)	2 (4.00%)	
MPA plus CNI	4 (12.50%)	2 (4.00%)	
No reduction	26 (81.24%)	41 (82.00%)	
Mortality, n (%)	10 (31.25%)	4 (8.00%)	**

BMI, body mass index; COVID-19, coronavirus disease; HLA, human leukocyte antigen; HBV, hepatitis B virus; HCV, hepatitis C virus; IL-2, interleukin-2; r, ATG, anti-thymocyte immunoglobulin; RTX, rituximab; IS, immunosuppressant; FK, tacrolimus; CsA, cyclosporine; MPA, mycophenolic acid; CNI, calcineurin inhibitor. *P<0.05; **P<0.01.

### 3.2 Univariate and multivariate analyses of death in severe COVID-19

To further explore the risk factors for death in severe COVID-19, we used the univariate and multivariate logistic regression model to analyze the risk factors (**Table 2**). The univariate analysis revealed that co-infection with pulmonary aspergillosis (OR=5.24, 95% CI: 1.3420.51, *P*=0.017), pre-infection AF insufficiency (OR=5.23, 95% CI: 1.4718.54, *P*=0.010), maintenance of a mycophenolic acid (MPA) dose >1500 mg/day before infection (OR=7.000, 95% CI: 1.6929.04, *P*=0.007), and the use of mechanical ventilation (OR=4.33, 95% CI: 1.3114.35, *P*=0.016) were significant risk factors death in severe COVID-19. We included covariates with *P*<0.1 in the univariate analysis and significant clinical variables in the multivariate analysis. The results showed that only AF insufficiency (OR=6.96, 95% CI: 1.4633.18, *P*=0.015) and maintenance of an MPA dose >1500 mg/day before infection (OR=7.59, 95% CI: 1.0853.20, *P*=0.041) were significant risk factors for death in severe COVID-19 infection, whereas the use of nirmatrelvir/ritonavir before severe COVID-19 diagnosis (OR = 0.21, 95% CI: 0.041.00, *P*=0.049) was a protective factor. Concomitant *Aspergillus* infection (OR=3.70, 95% CI: 0.7119.15, *P*=0.12) and mechanical ventilation (OR=2.76, 95% CI: 0.5713.49, *P*=0.21) were not significant risk factors (**Table 2**).

**Table 2:** Univariate and multivariate analyses of death in patients with severe COVID-19.

Variable	Univariate analysis			Multivariate analysis		
	Odds ratio	95% confidence interval	*P*-value	Odds ratio	95% confidence interval	*P*-value
Age (years)						
≤60	Reference					
>60	5.15	0.3087.75	0.26			
Time since transplantation at the end of follow-up in years						
≤5	Reference					
>5	0.50	0.131.97	0.32			
Donor type						
Living	Reference					
Deceased	1.13	0.284.55	0.87			
HLA mismatch						
≤3	Reference					
>3	1.55	0.445.45	0.50			
Cardiovascular disease						
No	Reference					
Yes	1.413	0.3385.908	0.64			
Chronic lung disease						
No	Reference					
Yes	5.15	0.3087.75	0.26			
Diabetes						
No	Reference					
Yes	2.44	0.748.07	0.15			
HBV infection						
No	Reference					
Yes	1.57	0.495.07	0.45			
HCV infection						
No	Reference					
Yes	2.32	0.618.86	0.22			
Complete vaccination						
No	Reference					
Yes	0.33	0.042.72	0.30			
Induction agent						
No	Reference					
Yes	0.81	0.087.87	0.86			
Using nirmatrelvir/ritonavir before severe COVID-19 infection						
No	Reference					
Yes	0.20	0.050.79	*	0.15	0.030.72	*
Using nirmatrelvir/ritonavir after severe COVID-19 infection						
No	Reference					
Yes	0.95	0.303.04	0.93			
Discontinuation of IS before severe COVID-19 infection						
No	Reference					
Yes	0.51	0.161.63	0.26			
Maintain MPA dose of >1500 mg per day						
No	Reference					
Yes	7.00	1.6929.04	**	7.59	1.0853.20	*
Concomitant *Aspergillus* infection						
No	Reference					
Yes	5.24	1.3420.51	*	3.70	0.7119.15	0.12
Mechanical ventilation						
No	Reference					
Yes	4.33	1.3114.35	*	2.76	0.5713.49	0.21
Allograft function insufficiency before COVID-19 infection						
No	Reference					
Yes	5.23	1.4718.54	*	6.96	1.4633.18	*

BMI, body mass index; COVID-19, coronavirus disease; HLA, human leukocyte antigen; HBV, hepatitis B virus; HCV, hepatitis C virus; IS, immunosuppressant; MPA, mycophenolic acid; CNI, calcineurin inhibitor. *P<0.05; **P<0.01.

### 3.3 Prognostic nomogram for death in severe COVID-19

**Figure 2:**
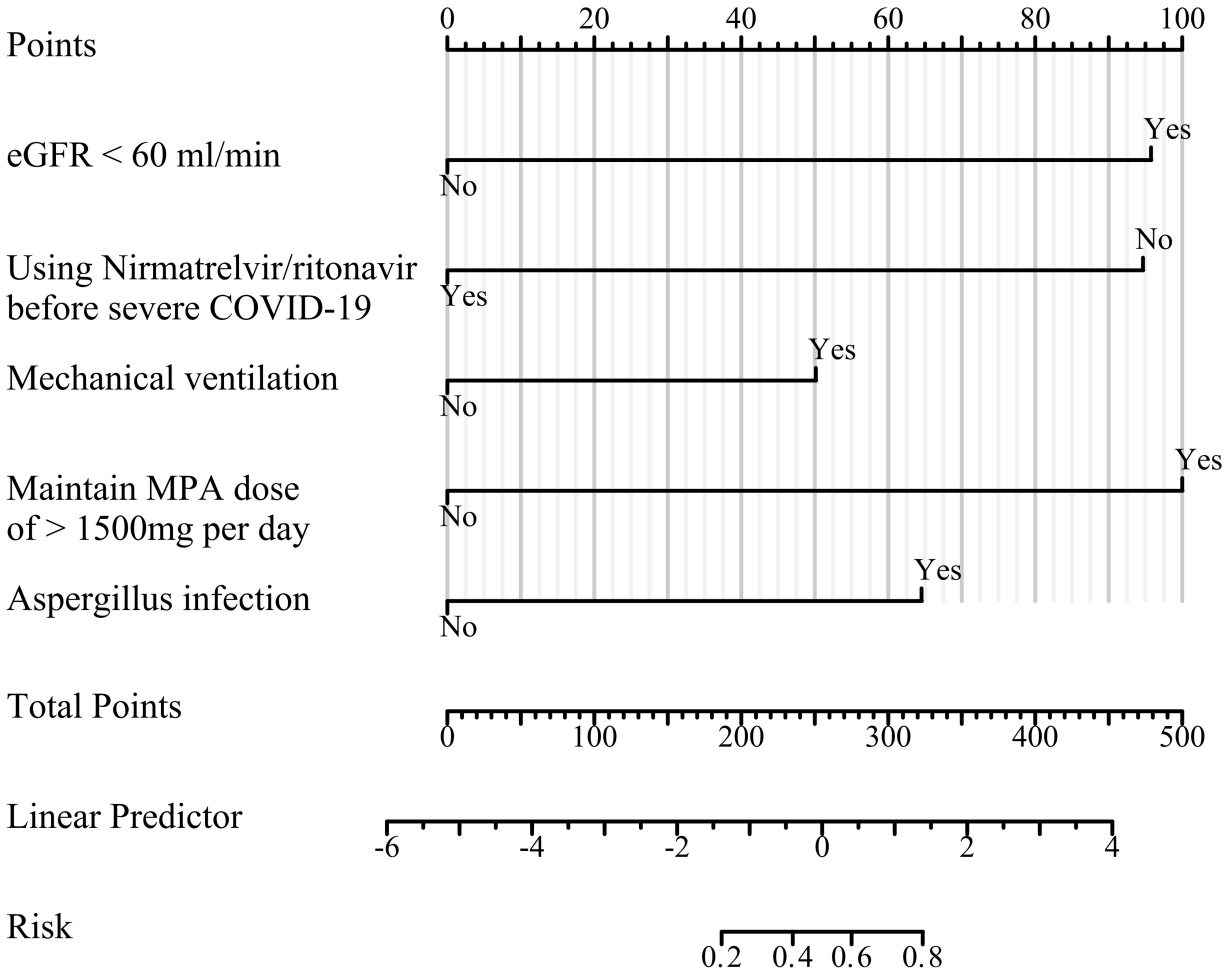
Prediction model nomogram. For each variable, the patients status value is plotted on the unique scale for that variable, and a vertical line is drawn from that location to the points line to determine a point value for that variable. The points for all variables are then added for a total point score.

Regarding the prognosis of patients with severe COVID-19, we generated a nomogram based on variables included in the multivariate model (**Figure 2**). Each variable was assigned a score ranging from 0 to 100, and the total scores of all variables were added to estimate mortality.

### 3.4 Impact of small-molecule antivirals on AF

**Figure 3:**
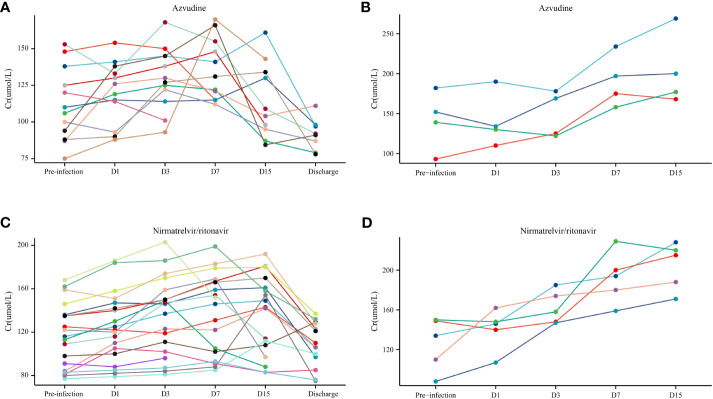
The allograft function (Cr) in patients with coronavirus disease (COVID-19) who used small-molecule antivirals. **(A)** Azvudine non-death group. **(B)** Azvudine death group. **(C)** Nirmatrelvir/ritonavir non-death group. **(D)** Nirmatrelvir/ritonavir death group.

During the study period, 16 KTRs received azvudine, whereas 24 received nirmatrelvir/ritonavir. Among the surviving KTRs, 29.03% (9/31) had pre-infection AF insufficiency, with 9.68% (3/31) and 19.35% (6/31) of them receiving azvudine and nirmatrelvir/ritonavir, respectively (**Figures 3A, C**). However, the results indicated that AF did not undergo irreversible damage following the administration of small-molecule antivirals (**Figure 3**).

### 3.5 Comparison of AF before and after COVID-19 infection

**Figure 4:**
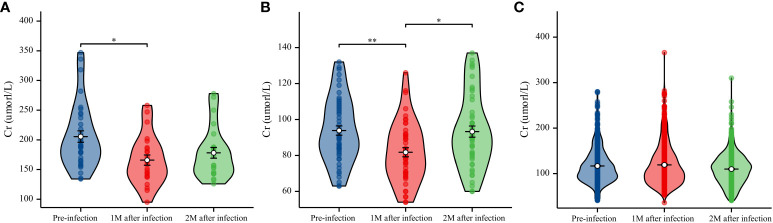
Data on creatinine (Cr) (μmol/L) levels were collected before infection and 1 and 2 months after infection. **(A)** Boxplot with bold line represents the median Cr (μmol/L) level in the decreased eGFR group. **(B)** Boxplot with bold line represents the median Cr (μmol/L) level in the normal eGFR group. **(C)** Boxplot with bold line represents the median Cr (μmol/L) level in the non-severe COVID-19 group. *P<0.05; **P<0.01.

Comparison of AF before and after COVID-19 diagnosis revealed that AF was significantly better 1 month after infection than before infection in the decreased eGFR (*P*<0.05) and normal eGFR (*P*<0.01) groups; however, there was no difference before and 2 months after infection (**Figures 4A, B**). We found no difference in AF before infection and one or two months after infection in the non-severe COVID-19 group (**Figure 4C**).

### 3.6 Complications after treatment for severe COVID-19

In the entire study population, no statistical difference was observed in complications related to allograft, including TCMR, urine BKPyV DNA load >8 log10, neo-HLA, neo-DSA before and after severe COVID-19 (2.44% [2/82] vs. 6.10% [5/82], *P*=0.44; 3.66% [3/82] vs. 9.76% [8/82], *P*=0.119; 3.66% [3/82] vs. 7.32% [6/82], *P*=0.49; 1.22% [1/82] vs. 2.44% [2/82], *P*=1.000; respectively). Similarly, there were no differences in complications unrelated to allograft, including lung superinfection, thrombus, and abnormal glucose levels (1.22% [1/82] vs. 1.22% [1/82], *P*=1.000; 2.44% [2/82] vs. 4.88% [4/82], P=0.677; 4.88% [4/82] vs. 7.32% [6/82], P=0.514; respectively). Notably, weight (61.79 [50.0965.84] vs. 56.71 [49.9660.93], P<0.001), hip circumference (92.90 [87.9197.61] vs. 91.13 [87.4494.97], P=0.03), and BMI (21.85 [20.6023.79] vs. 20.25 [18.5922.24], *P*<0.001) after severe COVID-19 were significantly lower than those before infection (**Table 3**).

**Table 3:** Other complications of kidney transplant recipients with severe COVID-19 before and after COVID-19 diagnosis.

Characteristics	Before COVID-19 diagnosis (n = 82)	After COVID-19 diagnosis (n = 82)	*P*-value
Related to allografts			
TCMR, n (%)	2 (2.44%)	5 (6.10%)	0.44
Urine BKPyV DNA load > 8 log^10^, n (%)	3 (3.66%)	8 (9.76%)	0.119
Neo HLA, n (%)	3 (3.66%)	6 (7.32%)	0.49
Neo DSA, n (%)	1 (1.22%)	2 (2.44%)	1.000
Not related to allografts			
Weight	61.79 (50.0965.84)	56.71 (49.9660.93)	***
Hip circumference	92.90 (87.9197.61)	91.13 (87.4494.97)	0.03
BMI	21.85 (20.6023.79)	20.25 (18.5922.24)	***
Lung superinfection, n (%)	1 (1.22%)	1 (1.22%)	1.000
Thrombus, n (%)	2 (2.44%)	4 (4.88%)	0.677
Abnormal glucose, n (%)	4 (4.88%)	6 (7.32%)	0.514

ALT, alanine aminotransferase; COVID-19, coronavirus disease; eGFR, estimated glomerular filtration rate. ***P<0.001.

## 4 Discussion

In this study, by constructing a multivariate logistic regression analysis, we found that pre-infection AF insufficiency was an independent risk factor for death in patients with severe COVID-19. AF insufficiency alters the homeostasis of fluid balance, electrolyte balance, and vascular tension, thereby exacerbating pulmonary infection (15). In addition, AF insufficiency can cause renal anemia and hypoproteinemia, leading to decreased immunity, which can increase the risk of death in an individual with COVID-19 (16). AF insufficiency can also lead to systemic damage, including dysfunction of the brain, heart, liver, and intestines (17, 18) and increased susceptibility to sepsis. Patients with chronic kidney disease and renal insufficiency have a significantly increased risk of death from severe COVID-19 (19), which is 10 times higher than that of patients with healthy renal function (20). This phenomenon was consistent in the KTRs. Nevertheless, our findings are not entirely consistent with the conclusions of a multicenter retrospective study (21). In contrast to previous studies, our analysis was based on pre-infection AF insufficiency, which was not affected by the SARS-CoV-2 infection. Therefore, for KTRs with pre-infection AF insufficiency, close attention should be paid to the changes following COVID-19. Interestingly, using nirmatrelvir/ritonavir before severe COVID-19 diagnosis reduced the risk of death in KTRs because nirmatrelvir/ritonavir can effectively inhibit SARS-CoV-2 (22). In addition, maintaining an MPA dose >1500 mg/day before severe COVID-19 and comorbidity with *Aspergillus* infection were risk factors for death in KTRs with severe COVID-19, similar to the findings of some studies (22, 23).

The kidney is one of the most common target organs of SARS-CoV-2 infection, and the incidence of AKI is considerably higher in KTRs than in the general population (11, 12). This raises one of the most concerning topics: Will small-molecule antiviral drugs exacerbate COVID-19-induced renal injury? Before answering this question, we should consider arguments regarding whether coronaviruses directly attack allografts. Su et al. (3) reported that autopsies of patients with COVID-19 revealed viral particles in the tubular epithelium and podocytes of the kidneys, which are ACE2-expressing cells, suggesting that severe COVID-19 significantly impacts the kidneys. Conversely. Golmai et al. (24) performed kidney autopsies on patients with COVID-19 diagnosed with stage 2 or 3 AKI; however, SARS-CoV-2 was not detected by immunohistochemistry. If the virus does not attack the kidney directly via an inflammatory response, drug-induced renal injury resulting from antiviral medications can exacerbate its impact on the allograft. Nirmatrelvir/ritonavir and azvudine are recommended antiviral drugs for COVID-19 treatment. As a reverse transcriptase inhibitor, azvudine shortens the nucleic acid negative conversion time (25), and nirmatrelvir/ritonavir can effectively inhibit SARS-CoV-2, significantly reducing the viral load in patients and thus decreasing the risk of death (22). However, in severe COVID-19, the effects of small-molecule antiviral drugs are still unclear, with no data on whether they exacerbate AF impairment. In our study, we found that the use of two small-molecule antiviral drugs in KTRs with severe COVID-19 did not further worsen AF impairment. Similarly, Toussi et al. (26) demonstrated that the safety of nirmatrelvir/ritonavir in patients with renal impairment was similar to that of azvudine (27). Adverse reactions during COVID-19 treatment require further exploration in future studies.

Another question we sought to clarify is whether AF will recover after severe COVID-19. Interestingly, we observed an improvement in AF 1 month after treatment for severe COVID-19 compared with that before infection. This phenomenon was also reported in a study from Italy, in which hospitalized KTRs had better AF after discharge than before infection, with no difference in non-hospitalized KTRs (28). However, this study did not specify whether the phenomenon occurred in patients with non-severe or severe COVID-19. Our data show that this paradox typically occurs in KTRs with severe COVID-19. Calcineurin inhibitors (tacrolimus and cyclosporine) are associated with AF impairment in KTRs (29). Thus, discontinuing calcineurin inhibitors in KTRs with severe COVID-19 may improve AF (30). Additionally, Cr is related to systemic nutritional status, and severe COVID-19 is a systemic-wasting disease that often causes malnutrition, which explains the temporary improvement in AF (31). For example, in our study, BMI, hip circumference, and weight were significantly lower after severe COVID-19 treatment conclusion than before the infection. A slightly worse AF at 2 months than at 1 month after treatment for severe COVID-19 confirms our conjecture. In addition, the occurrence of allograft-related complications after infection, such as TCMR, BKPyV, neo-HLA, and neo-DSA, indicates that the improvement in AF was temporary.

Our study had some limitations. First, this was a retrospective study, which inevitably involved information bias and potential confounding factors. Second, this was a single-center study with a limited sample size, and the results require further verification using large-sample multicenter research. Third, we did not confirm the variant of SARS-CoV-2 infection by conducting a specific PCR but instead used an antigen test paper or SARS-CoV-2 PCR.

In conclusion, this is the first report of a correlation between pre-infection AF insufficiency and mortality in patients with severe COVID-19. Pre-infection AF was a good predictor of death in KTRs with severe COVID-19. Additionally, an MPA dose >1500 mg/day before infection, non-use of nirmatrelvir/ritonavir before severe COVID-19, use of mechanical ventilation, and co-infection with pulmonary aspergillosis were associated with death in KTRs with severe COVID-19. AF was improved after the treatment of severe COVID-19, whereas this effect was not detected in non-severe COVID-19.

## Data availability statement

The raw data supporting the conclusions of this article will be made available by the authors, without undue reservation.

## Ethics statement

The studies involving humans were approved by Ethics Committee of the Sichuan Provincial Peoples Hospital. The studies were conducted in accordance with the local legislation and institutional requirements. The participants provided their written informed consent to participate in this study. Written informed consent was obtained from the individual(s) for the publication of any potentially identifiable images or data included in this article.

## Author contributions

HL: Writing – original draft, Conceptualization, Data curation. JW: Data curation, Methodology, Writing – original draft, Funding acquisition. HY: Data curation, Writing – review & editing. QR: Data curation, Supervision, Writing – review & editing. YH: Data curation, Project administration, Writing – original draft, Funding acquisition.
